# Skinfolds compressibility and digital caliper’s time response in skinfold measurement in male and female young adults

**DOI:** 10.1080/15502783.2023.2265888

**Published:** 2023-10-04

**Authors:** Raquel Vaquero-Cristóbal, Ana Catarina-Moreira, Francisco Esparza-Ros, Carlos Barrigas, Mario Albaladejo-Saura, Filomena Vieira

**Affiliations:** a International Kinanthropometry Chair, UCAM Universidad Católica San Antonio de Murcia, Murcia, Spain; b Facultad de Deporte, UCAM Universidad Católica San Antonio de Murcia, Murcia, Spain; c Instituto Politécnico de Lisboa, H&TRC-Health & Technology Research Center, ESTeSL-Escola Superior de Tecnologia da Saúde de Lisboa, Lisboa, Portugal; d Instituto de Estudos Interculturais e Transdisciplinares, Fisiologia e Bioquímica do Exercício, Almada, Portugal; e University of Lisbon, Laboratory of Biomechanics and Functional Morphology, Interdisciplinary Centre for the Study of Human Performance (CIPER), Faculty of Human Kinetics (FMH), Lisbon, Portugal; f Faculty of Sport Sciences, University of Murcia, Murcia, Spain

**Keywords:** Skinfold thickness, anthropometry, body composition, response time, skinfold caliper, fat mass

## Abstract

**Background:**

The skinfold caliper reading of the skinfold thickness depends on its dynamic compressibility. This has led to the fact that, while it is indicated that skinfold readings should be taken when the reading is stable, there is no consensus on at what second the reading should be taken after the application of the skinfold caliper. The new Lipowise PRO digital skinfold caliper was used to analyze the evolution of skinfold readings under skinfold caliper pressure. The aim of the present investigation were: a) to analyze the evolution of the reading time of individual skinfolds when subjected to skinfold caliper pressure and when the skinfold reading reaches stability; b) to describe the physical behavior of skinfold tissues’ time response to skinfold caliper pressure, and to explore differences between sites and subjects’ skinfolds compressibility; and c) to analyze the sex differences in both the reading and the evolution of the skinfold over time.

**Methods:**

A descriptive cross-sectional design was followed with a convenience sample of 165 healthy young adults (79 males and 86 females), with eight skinfolds measured using the Lipowise PRO skinfold caliper. The Lipowise PRO skinfold caliper uses a programmable reading time allowing for the measurement of the skinfold’s thickness at a rate of 100 times per second, and monitoring skinfold behavior over the 3-second measurement period, thereby enabling the assessment of the tissue response to the constant force exerted by the skinfold caliper jaws.

**Results:**

All skinfolds showed statistical differences in terms of compressibility characteristics (*p* < 0.001). Significant differences were found between measurement time points for individual skinfolds and sum of skinfolds (*p* < 0.001–0.025). Stabilization being found depending on the skinfold measured from 1.5 seconds for biceps, subscapular, iliac crest, supraspinale, abdominal, and thigh skinfolds; 2.0 seconds for ∑6 and ∑8 skinfolds; and 2.5 seconds for triceps and calf skinfolds. It was observed an effect of sex on this issue (*p* < 0.001–0.030). More specifically, in the case of males, the supraspinale and abdominal skinfolds stabilized after 1.5 seconds; the calf skinfold and ∑6 and ∑8 skinfolds stabilized after 2 seconds; while the rest of the skinfolds did not stabilize until 3 seconds. In the case of females, no stabilization of the triceps skinfold was found, while the rest of the individual skinfolds and the ∑6 and ∑8 skinfolds stabilized from 1.5 seconds. A regression analysis indicated that skinfold thickness could be predicted based on measurement time in 50–77% of the cases (*p* = 0.001).

**Conclusion:**

A skinfold caliper application, using the digital caliper Lipowise PRO, of three seconds may be sufficient for achieving stability in the measurement and for obtaining the minimum value for most individual and sum of skinfolds. However, there are certain skinfolds that may require more time when performed on certain individuals, which vary according to sex.

## 1. Introduction

The assessment of body composition is a common and useful practice in the context of health and sports. Among the different methods for assessing body composition, anthropometry is one of the most widely used for field testing to estimate body adiposity [1,2], as it is low-cost, portable technique, which uses relatively simple measurement procedures and allows for fast data collection. The measurement of skinfold thickness, which comprises two layers of skin and subcutaneous adipose tissue, allows both the estimation of adipose tissue, by usingthe value of individual skinfolds, considered an indirect method; and the estimation of lipid mass through the use of different regression equations, which includesskinfold values to estimate body density, and from body density estimate the lipid mass, so it is considered a doubly indirect method [3,4].

However, the use of skinfold thickness and anthropometric equations to assess body composition have some limitations, as that, the compressibility of the skin and subcutaneous adipose tissue is constant is assumed [5]. But it is known that these assumptions do not always apply to all populations or subjects studied, resulting in potential sources of errors in the assessment of body composition [6].

Furthermore, the skinfold caliper reading of the skinfold thickness depends on its dynamic downward compressibility, i.e. the way tissues decrease in thickness as a consequence of pressure exerted by the skinfold caliper [5,7]. As a consequence of the above, in the manufacture of the skinfold calipers, standard conditions have been sought where the skinfold calipers exert an average upward pressure of around 10.0 g/mm^2^ and downward pressure between 7.51 and 8.67 g/mm^2^ [7–9], assuming that variations ≤2.0 g/mm^2^ between upward and downward pressure are trivial and have historically been generalized as tolerable [7,8]. It is worth noting that most of these factories also carry out pressure measurements under static conditions, and very few data have assessed the pressure exerted by the skinfold caliper under dynamic conditions, even though this is the real situation when the skinfold caliper is applied [5,7]. Because of the above, the inherent physical, mechanical and functional specificity of each type of skinfold caliper may makes it impossible to use them interchangeably, and it is important that there is a similar structure between skinfold calipers to improve agreement between them [7].

The anthropometrist’s experience, noncompliance with standardized measurement procedures [10] can also interfere with the accuracy of the measurements obtained. In order to reduce this possible source of error, International Society for the Advancement of Kinanthropometry (ISAK) recommends that the reading of skinfold values should be made two seconds after the application of the skinfold caliper [5,10,11], while other protocols propose the reading of values after three seconds [12,13] and Bini et al. [14] suggested that in the case of having to carry out the evaluations quickly, the small change that occurs from 0.33 seconds onwards could be assumed, although they indicate that it would be possible to wait for 2–3 seconds as in other protocols for the value to be completely stable. However, even when adhering to a standard reading time, there may be different skinfold caliper readings for the same skinfold thickness, as a result of different degrees of static tissue compressibility [15].

In recent years, new opportunities for assessing skinfold compressibility in a direct, real-world application have emerged with the advent of the digital skinfold caliper, which introduces new functionalities, due to the combination of mechanical, electronic, and software innovations. The Lipowise PRO skinfold caliper from Lipowise, which originated from the Adipsmeter prototype (Lipotool, Portugal) [16], stands out in this range. Previous studies have shown that Adipsmeter prototype was a very accurate instrument [17] and that Lipowise PRO is a valid skinfold caliper for skinfold measurement in comparison to traditional dial skinfold calipers [18]. The advantages of Lipowise PRO are that it, when compared to traditional dial skinfold calipers, provides a faster assessment that is less influenced by the human factor, as it automatically acquires and analyzes data through a dedicated mobile application, also allowing the assessment of tissue compressibility made possible by its innovative technology of measuring skinfold thickness which takes 100 data per second, while making available a graphical illustration of the tissue response to constant force exerted by the skinfold caliper [13]. Thus, digital skinfold calipers can be used as a tool for the study of skinfold compressibility variation as a function of time elapsed after skinfold caliper application.

However, the few previous studies that have analyzed the dynamic evolution of tissue compressibility are inconclusive, showing differences in the evolution of the skinfold [13,14], in addition to the fact that they have used very small and unrepresentative samples of the adult population [13] or athletes [14]. Furthermore, these previous studies did not take into consideration that the compressibility of skinfolds is highly variable, and dependent on factors such as sexual dimorphism, age, location of skinfold, skin tension and thickness, distribution of connective tissue and blood vessels, and nutritional and hydration status [6,14,19,20]. A much debated question is whether there is an influence of other factors such as menstrual cycle or oral contraceptive cycle on measures of skinfolds, with recent studies finding that assessment could be done without considering these factors [21,22].

Therefore, the aims of the present investigation were: a) to analyze the evolution of the reading time of individual skinfolds when subjected to skinfold caliper pressure and when the skinfold reading reaches stability; b) to describe the physical behavior of skinfold tissues’ time response to skinfold caliper pressure, and to explore differences between sites and subjects’ skinfolds compressibility; and c) to analyze the sex differences in both the reading and the evolution of the skinfold over time.

## 2. Materials and methods

### 2.1. Experimental design

A descriptive cross-sectional design was followed, in accordance with the STROBE guidelines [23]. The PRESENT 2020 checklist was followed to write this manuscript [24]. The study was conducted both in the regions of Murcia (Spain) and Lisbon (Portugal) with a convenience sample.

All participants were volunteers and signed an informed consent form before starting the study. The privacy rights of human subjects were always observed. The study design, protocols and procedures followed the Code of Ethics of the World Medical Association (Declaration of Helsinki) for experiments involving humans and were approved by the Ethics Committees of the Faculty of Sport from the Catholic University of San Antonio – Murcia (CE012109) and of the Faculty of Human Kinetics from the University – Lisbon (CEFMH 10/2021).

### 2.2. Participants

The sample size was calculated with RStudio 3.15.0 software (RStudio Inc., Boston, MA, USA). The significance level was set at α = 0.05and the standard deviation (SD) was established based on the ∑8 skinfolds from previous studies (SD = 35.46) [18]. With an error (d) of 5.5 mm in the ∑8 skinfolds, the required sample was 165 subjects. Moreover, the calculation used to establish the required sample for each sex group was made using the ∑8 skinfolds standard deviation from research with samples of similar characteristics (SD = 27.35 for males; SD = 28.28 for females) [4]. With an error (d) of 6.0 mm in the ∑8 skinfolds in the case of the male population, and 6.2 mm in the case of the female population, the required sample was 79 subjects per group.

A total of 165 healthy young adults, 79 males (age = 21.81 ± 2.68 years old; stretch stature = 177.38 ± 19.24 cm; body mass = 67.79 ± 9.86 kg) and 86 females (age = 22.31 ± 3.72 years old; stretch stature = 165.08 ± 6.17 cm; body mass = 57.80 ± 6.97 kg) were included in the present study. To be considered eligible for the study, the participants had meet the following criteria: 1) Be caucasian, 2) Be aged between 18 and 25 years old, 3) Have a body mass index (BMI) between 18.5 kg.m^2^ and 24.9 kg.m^2^, 4) Not have any disease that could affect body fat, 5) Not have taken hormonal or corticosteroid treatment in the three months prior to the evaluation, and 6) For female participants, be between the 8th and 21st days of the menstrual cycle. Participants were excluded if: 1) Within 24 hours prior to the measurement session, had done vigorous physical exercise (or 12 hours in case of moderate exercise), 2) Had consumed products with diuretic properties within 24 hours prior to the measurement session, 3) Had eaten a heavy meal 24 hours prior to the measurement session, or 4) Had any injury that compromised the application of the measurement protocol [18].

### 2.3. Measurements

Basic measurements (body mass and stretch stature) and eight skinfolds (triceps, subscapular, biceps, iliac crest, supraspinale, abdominal, thigh and calf skinfolds) were obtained according to the ISAK guidelines [10] by three level 3 and two level 4 anthropometrists accredited by the ISAK. The mean intra-evaluator technical error of measurement (TEM) was 0.01% in the basic measurements, and 1.15% in skinfolds, and the mean inter-evaluator TEM was 0.04% in the basic measurements and 2.34% in skinfolds.

Body mass was measured to the nearest 0.1 kg with a digital SECA 878 scale (SECA, Hamburg, Germany) and stretch stature to the nearest 0.1 cm with a portable SECA 217 stadiometer (SECA, Hamburg, Germany), both measurements were obtained with participants barefoot and wearing minimal clothes. The eight skinfolds were measured with the digital Lipowise PRO skinfold caliper (Wisify, Porto, Portugal) to the nearest 0.1 mm. The Lipowise PRO device has been shown a downward pressure of 7.95 ± 0.19 g/mm^2^ [25]. The Lipowise PRO skinfold caliper uses a programmable reading time with the Lipowise Legacy software (Wisify, Portugal) allowing for the measurement of the skinfold’s thickness at a rate of 100 times per second, and monitoring skinfold behavior over the 3-second measurement period, thereby enabling the assessment of the tissue response to the constant force exerted by the skinfold caliper jaws. To assess adipose tissue compressibility the readings provided by the skinfold caliper in six measurement time points, 0.5s, 1s, 1.5s, 2s, 2.5s and 3s, were considered.

BMI (kg/m^2^), the sum of six skinfolds (triceps, subscapular, supraspinale, abdominal, thigh and calf) (mm), and the sum of eight skinfolds (triceps, subscapular, biceps, iliac crest, supraspinale, abdominal, thigh and calf) (mm), were calculated based on the anthropometric measurements.

To assess hydration status, the researchers provided participants with sterilized containers to collect a sample of urine as close as possible to the time of measurement, which was discarded by them at the end of measurement session. The urine color was determined simultaneously by two researchers in a well-lit room, by placing the urine sample container next to a color chart [26]. Each color of the chart was assigned a number from 1 to 8, with 1 corresponding to the lightest color and 8 to the darkest color, following the codification proposed of Armstrong [26].

### 2.4. Protocol

For each subject, the full set of anthropometric measurements were performed in a single day, from 8 a.m. to 2 p.m., in a private room with a comfortable and standardized temperature. The measurement protocol always began with marking anthropometric landmarks, followed by the measurement of basic measurements of body mass and stretch stature,and measurement of skinfolds.

Furthermore, the participants’ hydration status was assessed during the measurement session.

Lastly, the participants were asked to provide information on basic demographics, diseases that could affect body fat, hormonal or corticosteroid treatments and menstrual cycle phase.

### 2.5. Statistical analysis

The normality of the distribution was verified with the Kolmogorov-Smirnov test. Asymmetry and kurtosis were also verified. As all the variables included in the analysis followed a normal distribution, parametric statistical tests were performed. A descriptive analysis was performed for all the variables included. A repeated measurements MANCOVA test was performed to analyze the differences between the different measurement time points, and the covariable “sex” was included to verify its effect on the results obtained. A post hoc Bonferroni adjustment was used to analyze the pairwise comparison of the measurement time points. According to previous research, the lowest skinfold thickness (SL), the first moment (TL) in which the lowest value is reached, the 110% value of the skinfold thickness of point L (SH), and the time (TH) corresponding to the first moment in which that measurement is obtained, and the skinfold thickness (SS) measured at the maximum TH + 2 standard deviations, were calculated [14]. Finally, a regression analysis between the skinfolds thicknesses and the time of the measurement was performed. The SPSS (v.23, IBM, USA) software was used to perform the statistical analysis, calculate normality, and the MANCOVA test. The significance level was set a priori at α = 0.05.

## 3. Results

**Figure 1.**
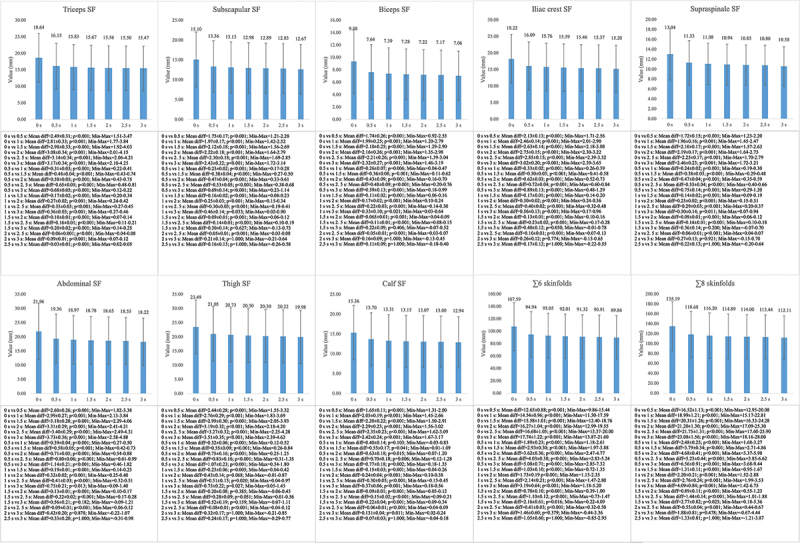
Mean differences between the measurement time points in the complete sample. SF= skinfold.

**Figure 2.**
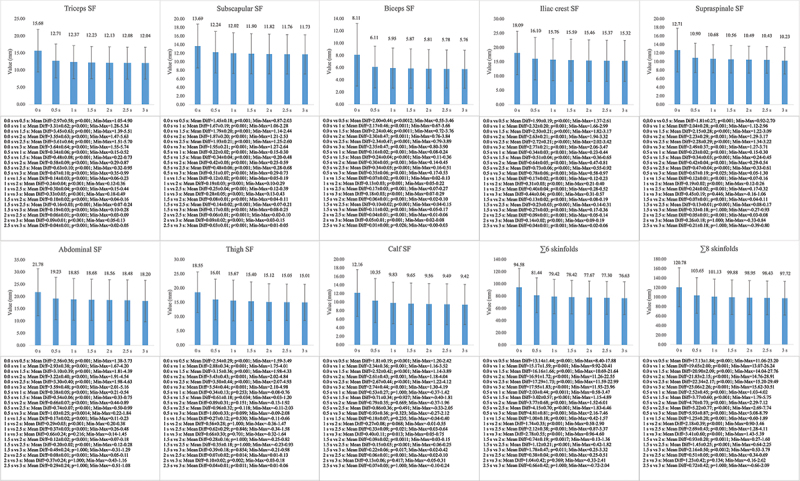
Mean differences between the measurement time points in the male sample. SF= skinfold.

**Figure 3.**
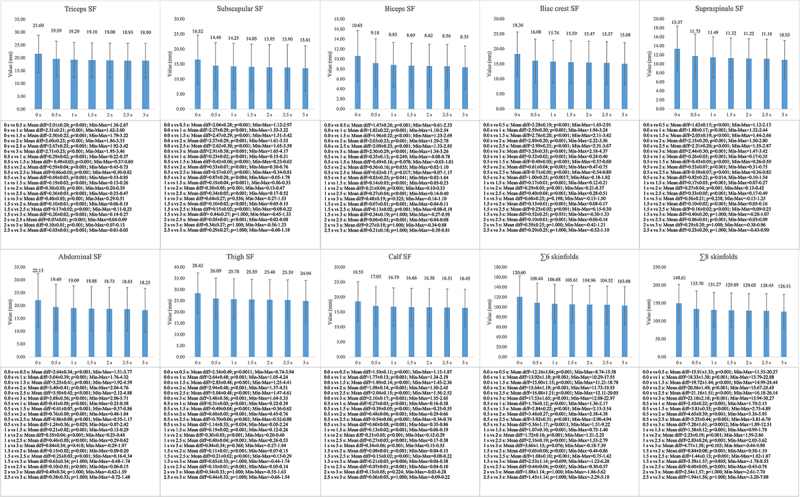
Mean differences between the measurement time points in the female sample. SF= skinfold.

The mean differences and standard deviations between the measurement time points for the triceps, subscapular, biceps, iliac crest, supraspinale, abdominal, thigh and calf skinfolds, and the ∑6 and ∑8 skinfolds, are shown in Figures 1–3. When the repeated measurements MANCOVA was performed for the complete sample, intra-subject differences were observed in all the variables included (F = 67.17–242.64; *p* < 0.001; η2p = 0.50–0.78). The Bonferroni pairwise comparison analysis of the general sample can be observed in Figure 1. In the case of the triceps and calf skinfolds, significant differences were observed between all the measurement time points analyzed, with respect to the final value (Mean difference (MD) = 0.03–3.17; *p* < 0.001–0.011), except in the comparison between the 2.5s measurement time point and the final value in the calf skinfold. For the other skinfolds, no significant differences were found between the values at the 1.5s, 2s, 2.5s measurement time points and the final value (MD = 0.11–0.42; *p* = 0.050–1.000) (Figure 1). For the ∑6 and ∑8 skinfolds, statistically significant differences were observed, except for the measurement time points 2.0s and 2.5s with respect to the final value (MD = 0.41–23.08; *p* < 0.001–0.025) (Figure 1).

When the effect of the covariable sex was analyzed, it was observed that it had an effect on the differences found in the triceps, subscapular, biceps, iliac crest, supraspinale, and abdominal skinfolds and in the ∑6 and ∑8 skinfolds (F = 4.92–33.21; *p* < 0.001–0.030; η2p = 0.07–0.33). With respect to this result, an analysis of the differences, by dividing the sample by sex, was performed.

Differences were observed in the male sample in all the variables analyzed (F = 27.60-192-96; *p* < 0.001; η2p = 0.45–0.85). The results of the Bonferroni pairwise comparison of the male sample can be observed in Figure 2. All the skinfolds showed significant differences between the different measurement time points (MD = 0.04–3.59; *p* < 0.001–0.034), except for the supraspinale and abdominal skinfolds between the 1.5s, 2s and 2.5s points and the final value, and the calf skinfold between the 2s and 2.5s points and the final value. Regarding the ∑6 and ∑8 skinfolds, differences were observed between all the measurement time points (MD = 0.38–23.06; *p* < 0.001–0.017), except for the 2s and 2.5s points and the final value.

Differences were also observed between all the variables analyzed in the female sample (F = 53.36–156.98; *p* < 0.001; η2p = 0.62–0.83). The results of the Bonferroni pairwise comparison of the female sample can be observed in Figure 3. The triceps skinfold results showed differences between all the measurement time points (MD = 0.03–3.88; *p* < 0.001). Statistical differences between the other skinfolds analyzed and the ∑6 and ∑8 skinfolds were observed (MD = 0.07–23.10; *p* < 0.001–0.041), except between the 1.5s, 2s and 2.5s points and the final value.

Table 1 shows the compressibility characteristics of the skinfolds analyzed. The thigh skinfold showed the highest values (SH = 20.95–27.86; SL = 19.04–25.33) in the overall sample and in the female population, while in the male population, the highest value was observed in the abdominal skinfold (SH = 16.80; SL = 15.27). The skinfold with the lowest values was the biceps (SH = 5.33–9.03; SL = 4.84–8.21). The skinfold that took the shortest time to stabilize the minimum value was the biceps in all cases (TL = 2.16–2.43), while the skinfold that took the longest was the iliac crest skinfold in the general and female samples (TL = 3.00), and the abdominal skinfold in the male sample (TL = 2.90). All skinfolds showed statistical differences between them in terms of compressibility characteristics (*p* < 0.001).

**Table 1.** Skinfolds thickness and time response.

General sample					
Skinfolds	S_H_ (mm)	S_L_ (mm)	S_S_ (mm)	T_H_ (s)	T_L_ (s)
Triceps	16.01±7.54	14.55±6.98	15.58±7.45	0.12±0.07	2.77±0.33
Subscapular	12.79±5.99	11.63±5.62	12.58±5.96	0.16±0.01	2.88±0.35
Biceps	7.27±4.53	6.61±4.16	7.05±4.43	0.18±0.08	2.22±0.29
Iliac crest	15.57±7.26	14.15±6.73	15.23±7.18	0.21±0.06	3.00±0.28
Supraspinale	11.11±6.39	10.10±6.03	10.89±6.39	0.17±0.07	2.93±0.26
Abdominal	18.12±9.18	16.47±8.22	17.80±9.01	0.15±0.07	2.95±0.24
Thigh	20.95±10.63	19.04±9.61	20.38±10.39	0.12±0.07	2.88±0.22
Calf	13.24±7.50	12.03±7.07	12.85±7.37	0.11±0.08	2.29±0.20
Mean	14.38±7.38	13.07±6.80	14.04±7.27	0.15±0.06	2.72±0.27
p value	<0.001	<0.001	<0.001	<0.001	<0.001
Male sample					
Skinfolds	S_H_ (mm)	S_L_ (mm)	S_S_ (mm)	T_H_ (s)	T_L_ (s)
Triceps	11.27±4.80	10.24±4.22	10.81±4.48	0.19±0.11	2.76±0.27
Subscapular	11.24±4.89	10.22±4.41	11.11±4.77	0.10±0.01	2.86±0.31
Biceps	5.33±3.84	4.84±3.42	5.21±3.76	0.22±0.09	2.43±0.24
Iliac crest	14.43±7.07	13.12±6.52	14.18±6.96	0.18±0.12	2.88±0.32
Supraspinale	9.96±6.02	9.05±5.65	9.73±5.86	0.15±0.07	2.87±0.33
Abdominal	16.80±8.37	15.27±7.78	16.20±8.24	0.27±0.12	2.90±0.31
Thigh	13.92±7.26	12.65±5.52	13.58±6.51	0.24±0.11	2.87±0.30
Calf	8.17±4.61	7.43±4.07	8.17±4.64	0.24±0.12	2.77±0.29
Mean	11.39±5.86	10.35±5.20	11.12±5.65	0.20±0.09	2.79±0.30
p value	<0.001	<0.001	<0.001	<0.001	<0.001
Female sample					
Skinfolds	S_H_ (mm)	S_L_ (mm)	S_S_ (mm)	T_H_ (s)	T_L_ (s)
Triceps	20.51±7.08	18.65±6.62	20.23±7.15	0.08±0.01	2.98±0.33
Subscapular	14.08±6.64	12.80±6.35	14.03±6.62	0.16±0.03	2.82±0.29
Biceps	9.03±4.45	8.21±4.00	8.83±4.38	0.25±0.06	2.16±0.22
Iliac crest	16.42±7.37	14.93±6.83	15.95±7.19	0.34±0.05	3.00±0.37
Supraspinale	11.99±6.67	10.90±6.21	11.84±6.75	0.21±0.02	2.97±0.34
Abdominal	19.16±9.58	17.42±8.32	18.82±9.28	0.30±0.08	2.62±0.27
Thigh	27.86±9.36	25.33±8.63	27.75±9.40	0.06±0.01	2.97±0.31
Calf	17.76±7.12	16.15±6.81	17.61±6.98	0.10±0.01	2.20±0.24
Mean	17.10±7.28	15.55±6.72	16.88±7.22	0.19±0.03	2.72±0.30
p value	<0.001	<0.001	<0.001	<0.001	<0.001

SL: Lowest of the 120 measurements; S_H_: 110% of point L; S_S_: Skinfold thickness measured at the maximum value of TH+2SD; T_L_: First measurement time point where the minimum skinfold thickness was measured; T_H_: First time point where 110% of point L was measured.

Regarding the regression analysis performed between skinfold thickness and measurement time (Table 2), it was observed that the skinfold thickness value could be predicted depending on the measurement time (s) in 50–77% of the cases (*p* = 0.001).

**Table 2.** Regression analysis related to the skinfold thickness and the measuring time.

Variable	R^2^	p value	Included Independent Variables	SC	Equation
General sample					
Triceps sf	0.62	0.001	Time	−0.79	Triceps Sf = 15.566–0.004*(t*100)
Subscapular sf	0.70	0.001		−0.83	Subscapular Sf = 12.55–0.003*(t*100)
Biceps sf	0.62	0.001		−0.79	Biceps Sf = 7.230–0.002*(t*100)
Iliac crest sf	0.77	0.001		−0.88	Iliac crest Sf = 15.479–0.005*(t*100)
Supraspinale sf	0.70	0.001		−0.83	Supraspinale SF = 10.944–0.003*(t*100)
Abdominal sf	0.66	0.001		−0.81	Abdominal Sf = 17.884–0.005*(t*100)
Thigh sf	0.76	0.001		−0.87	Thigh Sf = 20.460–0.005*(t*100)
Calf sf	0.57	0.001		−0.76	Calf Sf = 12.869–0.003*(t*100)
∑8 sf	0.71	0.001		−0.84	∑8 Sf = 112.973–0.03*(t*100)
Males					
Triceps sf	0.55	0.001	Time	−0.74	Triceps Sf = 11.078–0.003*(t*100)
Subscapular sf	0.69	0.001		−0.83	Subscapular Sf = 10.946–0.002*(t*100)
Biceps sf	0.64	0.001		−0.80	Biceps Sf = 5.408–0.002*(t*100)
Iliac crest sf	0.70	0.001		−0.84	Iliac crest Sf = 14.292–0.004*(t*100)
Supraspinale sf	0.65	0.001		−0.81	Supraspinale SF = 9.854–0.003*(t*100)
Abdominal sf	0.62	0.001		−0.79	Abdominal Sf = 16.548–0.004*(t*100)
Thigh sf	0.76	0.001		−0.87	Thigh Sf = 13.95–0.004*(t*100)
Calf sf	0.66	0.001		−0.81	Calf Sf = 8.352–0.003*(t*100)
∑8 sf	0.70	0.001		−0.84	∑8 Sf = 90.428–0.026*(t*100)
Females					
Triceps sf	0.68	0.001	Time	−0.82	Triceps Sf = 19.784–0.004*(t*100)
Subscapular sf	0.65	0.001		0.81	Subscapular Sf = 13.955–0.004*(t*100)
Biceps sf	0.55	0.001		−0.74	Biceps Sf = 8.943–0.003*(t*100)
Iliac crest sf	0.77	0.001		−0.88	Iliac crest Sf = 16.513–0.006*(t*100)
Supraspinale sf	0.69	0.001		−0.83	Supraspinale SF = 11.914–0.004*(t*100)
Abdominal sf	0.65	0.001		−0.81	Abdominal Sf = 19.096–0.006*(t*100)
Thigh sf	0.74	0.001		−0.86	Thigh Sf = 26.906–0.005*(t*100)
Calf sf	0.50	0.001		−0.70	Calf Sf = 12.869–0.003*(t*100)
∑8 sf	0.70	0.001		−0.84	∑8 Sf = 134.216–0.034*(t*100)

Sf: Skinfold; t: time in seconds.

## 4. Discussion

The aims of the present investigation were to analyze the evolution of the reading time of individual skinfolds when subjected to skinfold caliper pressure and when the skinfold reading reached stability, describe the physical behavior of the skinfold tissues’ time response to skinfold caliper pressure, and to explore differences between sites and subjects’ skinfolds compressibility. The results showed that while the triceps and leg skinfolds could be read starting at 2.5 seconds, the rest of the skinfolds stabilized at 1.5 seconds. The sum of skinfolds stabilized from 2 seconds onwards. In fact, to find the time point at which the lowest values could be found, an analysis was performed of the values every hundredth of a second, with the lowest values found between 2.22 seconds and 3.00 seconds for the overall sample. This is important, considering that a skinfold is defined as the minimum double layer of subcutaneous adipose tissue offering parallel surfaces plus the skin adjacent to this fat [10], which explains why the minimum value is relevant in the measurement of skinfolds. There has been much debate as to how much time is needed for a skinfold reading to be stable [5,10,12,14]. In previous studies, skinfolds have shown a significant decrease in a short period of time when the skinfold caliper was applied to compress the adipose tissue, after which the measurements became stable, without significant differences in the reading of the skinfolds [13,14], with similar results also found in the present research study. Not surprisingly, in the present study it was found that in a high percentage of the sample (between 50 and 77% of the cases), the skinfold thickness changed with time t according to the equation: y = y0 + a⁄(b + tn). These results coincide with those found in previous research conducted in a young male athlete population. In fact, in that study, a much higher percentage of compliance with this equation was found (approximately 99%) [14]. In light of the results of the present research, this equation also explains the evolution of skinfolds in the much more general population.

The decrease observed by the skinfold value over time after the use of the skinfold caliper could be due to several factors, including the fact that the skinfold caliper exerts a certain uniform pressure across the entire application area, which is standardized at 10 g/mm^2^ in upscale and 7.51–8.67 g/mm^2^ in downscale [7–9], with both fat and blood vessels showing changes in their arrangement when subjected to pressure [8]; although the physical, mechanical and functional specificity of each type of skinfold caliper may generate variations in the pressure exerted by the skinfold caliper [7]. The differences in the time it takes for values to stabilize between skinfolds could be due to both the skin and adipose tissue having different thicknesses and structures across the body [6,27]. The results from the present investigation are consistent with those found in a pilot study conducted with only 10 subjects, including both males and females, with the results for the triceps skinfold and when it was found similar to the present investigation, with a stable reading obtained at 2.5 seconds [13]. However, there have been no previous studies that analyzed this matter in a significant sample or the evolution of the rest of the skinfolds. However, the results contrast slightly with those found in a previous study with a sample of young male athletes, which indicated that the time needed to obtain the minimum value in the skinfold assessment was 1.46 ± 0.34 seconds [14], as opposed to 2.72 ± 0.27 in the present investigation. This could be due to the differences between the samples, with the sample of the Bini study being smaller and more homogeneous, and the population of the present study being closer to the general young adult population who have larger skinfold values, which could be affecting the stabilization of the skinfolds [14]. Therefore, in general terms, in light of the present research, it could be decide that skinfolds can be read in the thrird second after application of the caliper, regardless of the intention of evaluating the sum of skinfolds or the individual skinfolds evolution, to be sure that the final value is stable and minimum.

Another important finding of the present investigation was that significant differences were found between skinfolds in the variables SH, SL, SS, TH and TL, confirming that the compressibility of each skinfold is different from that of the others. The results of the present investigation are consistent with those found in a previous study conducted with 36 adult male professional athletes, which is to date the only study that analyzed this question [14], allowing the results of this preliminary study to be extrapolated to a more general young adult population and to the female sex.

Another objective of the present research was to analyze the differences according to sex in both the reading and the evolution of the skinfolds over time. A relevant result was that sex had an effect on the time it took for the skinfolds of both the upper limb and the trunk, and also the sum of the skinfolds to stabilize. More specifically, in the case of men, the stability of most of the skinfolds might not be reached in these three seconds, except in the case of the supraspinale, abdominal, and leg skinfolds. However, the value of the sum of the skinfolds was found to be stable after two seconds. On the other hand, in the case of women, the values of all the individual skinfolds and the sum of the skinfolds were found to be stable after 1.5 seconds, except in the case of the triceps skinfold. With regard to the analysis of the evolution of the compressibility of the skinfolds as a function of sex, it was found that sex did not have an influence on the time at which the lowest value was found in the reading of the skinfold, with this being between 2.43 seconds and 2.90 seconds for the sample of men, and between 2.16 seconds and 3.00 seconds for the sample of women. Previous studies had hypothesized that the compressibility and stabilization of skinfolds might vary according to sex, given the influence of factors such as skin tension and thickness, distribution of connective tissue and blood vessels, and the distribution and characterization of fat mass [6,27,28]. However, thus far, there are no known studies that have analyzed this issue in real-life situations. Therefore, given the interesting results from this research, it is necessary for future studies to analyze the issue of compressibility and readability in men and women separately, and to measure beyond three seconds. Although it seems that the minimum thickness the skinfold has been reached at this time point, and that the skinfold could be stable, it might be necessary to extend the reading time to verify this, especially in some individual skinfolds.

The present study is not without limitations. Among them we find that the reading was limited to 3 seconds, following the maximum time proposed in anthropometric research and protocols in order to achieve skinfold stability and a reading of the minimum skinfold value [5,10,12,14]. Although in the present research it was found that this length of time could be sufficient in general terms, in some very particular cases it was not possible to analyze whether the value read at these three seconds would be significantly similar to the one that could be reached at a later reading, which would imply that the stability of the reading has been reached. Therefore, in future research, it would be convenient to extend the reading time of the skinfold caliper to corroborate the results of this research as the Lipowise PRO skinfold caliper as well as the other digital and dial skinfold calipers do not have a time limitation for the reading. Another limitation of the present study was the absence of other types of digital or dial skinfold calipers when taking measurements. As previous studies have shown that skinfold calipers may not be interchangeable [7,18], this is an important issue that limits the generalizability of the results. So, the findings of the present research are limited to the use of Lipowise PRO. Therefore, further research is needed to further explore this issue with other skinfold calipers and try to try to generalize the results found in the present study. Another limitation was the heterogeneity of the sample in terms of physical exercise habits, nutritional habits, etc. although this corresponds to the interest in analyzing a general population in order to obtain results that could be extrapolated to reality as much as possible.

In conclusion, a skinfold caliper application, using the digital caliper Lipowise PRO, of three seconds may be sufficient for achieving stability in the measurement and for obtaining the minimum value for most individual and sum of skinfolds. However, individual analysis of the triceps and iliac crest skinfolds for the general sample; triceps, subscapular, biceps, iliac crest and thigh skinfolds for men; and triceps and iliac crest skinfolds for women; may require more time when performed on certain individuals.
